# The relationship between sole curvature of roll over footwear and changes in gait

**DOI:** 10.1186/1757-1146-5-S1-O5

**Published:** 2012-04-10

**Authors:** Saeed Forghany, Christopher Nester, Barry Richards

**Affiliations:** 1 School of Health Sciences, University of Salford, UK; 2 Musculoskeletal Research Centre, School of Rehabilitation Sciences, Isfahan University of Medical Sciences, Iran

## Background

Footwear with a curved sole profile remains popular and there is evidence of its effects on gait and posture [1,2]. Existing literature describes how different styles of roll over footwear affect gait, although the effects of the precise radii of the curve of the sole on the gait rocker function and other reported effects have not been investigated. The aim of this study was to relate the radii of the soles of two roll over footwear products to their effects on walking.

## Materials and methods

Lower limb kinematic and GRF data was collected from twenty subjects during walking in four footwear conditions: flat control shoe, weighted flat control shoe, the new prototype rollover shoe and a MBT shoe. The static sole radii of all footwear was calculated for all and distinct parts of the sole, and correlated with radii of the gait rollover shapes as described by Hansen et al [3,4].

## Results

The radii of the foot–shoe roll-over shapes were significantly changed in response to different shoe conditions (p<0.001), but leg and thigh radii were not. The MBT shoes demonstrated a low positive correlation between the radius of foot-shoe roll-over shape and the static sole radii (whole sole) (r = 0.32; p = 0.04) and the radii of the heel area of the sole (r = 0.39; p = 0.01). The new prototype shoes showed no statistically significant correlations.

## Conclusion

The results of this study indicate that the static curve of the sole is not the main factor influencing gait. It appears that the extent to which the curved sole deforms dynamically during gait (i.e. shoe bending stiffness) influences the strength of the relationship between sole curvature and changes in gait.
